# Air Pollution, Health Shocks and Labor Mobility

**DOI:** 10.3390/ijerph19031382

**Published:** 2022-01-26

**Authors:** Yi Zhang, Tao Shi, Ai-Jun Wang, Qi Huang

**Affiliations:** 1 School of Business, Jiangsu Normal University, Xuzhou 221116, China; zy2021@jsnu.edu.cn; 2 Economics Institute, Henan Academy of Social Science, Fengchan Road 21, Zhengzhou 450002, China; 3 Economics School, Zhongnan University of Economics and Law, Nanhu Avenue 182, Wuhan 430073, China; 4 Zhengzhou Central Sub-Branch of People’s Bank of China, Shangwu Road 21, Zhengzhou 450000, China; huangqixl@163.com

**Keywords:** air pollution, health shocks, labor mobility, mediating effect, threshold effect

## Abstract

The health shocks caused by air pollution seriously interfere with people’s economic life. Based on the air pollution index and health shock index calculated by the principal component entropy weight method, this article analyzes the impact of air pollution on labor mobility, and adopts the mediation effect model to test the mediation effect of health shocks, using the threshold model to analyze the time and the health shocks threshold effect of air pollution on labor mobility. Its conclusions are as follows: (1) Air pollution has a negative impact on the net inflow of labor mobility, and the net inflow of labor mobility decreases between 24.9% and 44.7% on average for each unit increase in the health shocks of air pollution. (2) The impact of air pollution on labor mobility is all caused by health shocks; the health shocks are also an important factor influencing the decrease in the labor mobility supply across provinces, and the different health levels of the migrating individuals due to air pollution. (3) The health shocks of air pollution have a single-time threshold effect on labor mobility, and the health shocks of air pollution in China have intensified after 2010, confirming that China’s Lewis turning point was 2010. (4) The attraction effect of stable and higher regional real income will partially offset the repulsion effect of health shocks of air pollution on labor mobility, when the health-shocks index of air pollution exceeds the threshold value of 1.9873. Finally, the policy implications of the health shocks of air pollution on labor mobility are also formulated.

## 1. Introduction

**Figure 1:**
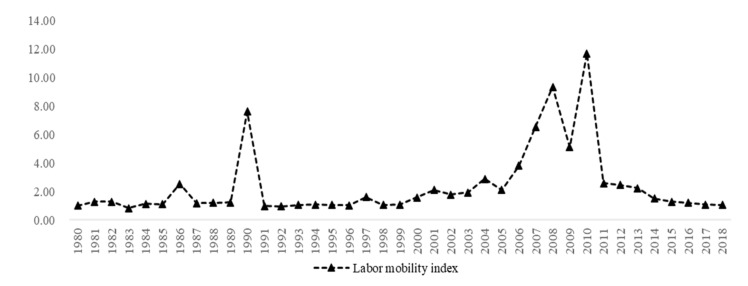
The trend of the labor mobility index and the annual average inflow of floating population in China from 1980 to 2018. Note: According to the calculation formula of net mobility rate in the Section 3.2.2, the annual results of each province were taken as the natural index, and then the annual average value of the whole country was calculated to represent the inflow index.

The environment, health and reasonable labor mobility are currently hot social topics. Since 1949, the scale of labor mobility has been unprecedented, and it has become an important factor for promoting social and economic development [1]. From 1982 to 2015, China’s labor mobility scale first increased and then decreased, with the total population migration across counties enlarging 4.1 times from 1982 to 2010, and decreasing 9.59% from 2010 to 2015 [2], becoming the main labor force for China’s economic development, especially in developed regions such as Guangdong, Shanghai and Beijing. Indeed, the characteristics of labor mobility in China can be divided into three stages. Firstly, the early stage of labor mobility (1949–1978). In this stage, only a small number of migrant workers can be migrated to another place, especially to the cities, and labor mobility is mainly based on the government’s planned policies to serve social stability and economic development. Secondly, the middle stage of labor mobility (1979–2010). In this stage, laborers begin to flee their regulated workplace for a better income, and the labor mobility scale across regions is growing. Both China’s labor mobility index and the annual average inflow of the mobile population are continuously increasing before 2010, as shown in Figure 1. Thirdly, the transition stage of labor mobility (after 2011). In this stage, the characteristics of health-type mobility appeared, and health demands come to be a more important factor influencing labor’s mobility.

**Figure 2:**
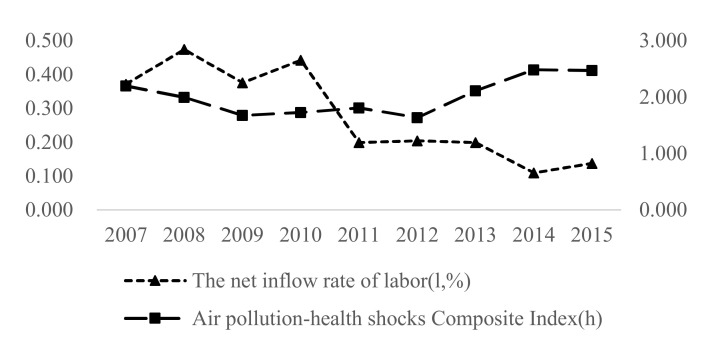
The trend of the net inflow rate of labor mobility and the air pollution-health shocks composite index from 2007 to 2015. Note: (1) The net inflow rate of labor mobility is calculated according to Formula (7) below, which is the average value of each year. (2) The air pollution-health shocks composite index is calculated in Section 3.2.2.

In the third stage, the deaths caused by air pollution increased. According to the Lancet’s “Global Burden of Disease Report 2019,” China’s PM2.5 caused 1.2 million early deaths and 25 million disabled-adjusted life-year losses in 2019. As the threat of air pollution and health damage continues to increase, the average annual labor mobility index (net labor inflow rate) in each province of China has continued to decline, and this indicates increased labor fleeing as shown in Figure 2(the graph is consistent with Zhang and Wang (2020) [3]). Based on this phenomenon, scholars believe that air pollution has a significant negative impact on labor mobility [4,5,6].

The negative relationship between air pollution and labor mobility refutes the view of surplus labor depletion under the “Lewis Turning Point” from the perspective of exogenous influence. Especially in the developed and eastern coastal regions in China, people decide their own labor supply on facts as health and social investment, the labor income, the leisure replacement and healthy life, and we can also see the counter-urbanization phenomenon known as “fleeing Beijing, Shanghai and Guangzhou” [6]. However, there are few empirical studies on the relationship between air pollution, health and labor mobility. Most studies do not provide direct evidence to confirm air pollution’s influence on labor mobility through damaging health, and focus especially on the healthy migration effect and the salmon bias effect after the self-selection of Latin immigrants (also known as the Hispanic paradox) and are limited to using the pollution amount or concentration and other related variables to construct indicators for indirect analysis [5,7]. The health paradox of Latino immigrants (the Hispanic paradox) refers to the fact that Latino immigrants with lower socioeconomic status in the United States who have limited access to healthcare generally have a higher health level than non-Hispanic whites [8,9]. Indeed, facing the health shocks caused by air pollution, the probability of labor migration across provinces is higher than that across counties, and health shocks on labor mobility are more sensitive in larger geographical regions [10]. Therefore, this article focuses on providing direct evidence of air pollution’s impact on labor mobility, anchoring the health shocks perspective in provincial samples. The potential contributions of this article are as follows: Firstly, using the principal component-entropy weight combination method, this article calculated the air pollution index and the health shocks effect index, and provided a direct provincial sample for empirical analysis. Second, using the mediation effect model, we found that labor will be impacted by health shocks when it suffers from air pollution. Labor mobility will be promoted to change migration decisions, and the inflow rate of labor mobility across provinces will decrease. These findings will provide additional evidence for the debate on the Lewis turning point and the exploration of the health paradox of Latin immigrants.

The remaining sections of this article are as follows: Section 2 is the literature review, summarizing the methods and conclusions about the relationship between air pollution, health shocks and labor mobility, and providing related hypotheses. Section 3 is the methodology, introducing the mediation effect method and data resources and description. Section 4 reveals the result, showing the impact of air pollution on labor mobility, and the mediation effect of health shocks and the threshold effect. Section 5 is the discussion, focusing on the study’s results and their limitations. Finally, in Section 6, the conclusions and policy implications are provided.

## 2. Literature Review

### 2.1. The Impact of Air Pollution on Health

Scholars have studied the relationship between different air pollutants and health, and found that air pollution has a significantly positive relationship with the death rate [11], the infant mortality rate [12], respiratory diseases [13], cardiovascular diseases [14], dermatology [15], ocular surface diseases (Jung et al., 2018) [16], neurological diseases [17], cancer [18] and other chronic diseases [19]. For example, Chay and Greenstone (2003) [20] asserted that with a 1% decrease in the total suspended particulate (TSP) the infant mortality would decrease 0.35−0.45%, and indicated a non-linear relationship between air pollution and infant mortality at the county level. Based on a sample of 88 large cities in the United States, Dominici et al. (2002) [21] argued that the mortality rate would increase 0.5% if the PM10 concentration rate on the previous day increased about 10 µg/m^3^, and indicated a linear relationship between concentration and response. Song et al. (2019) [14] further found that hospitalized patients with hypertension increased by 0.56%, 0.31%, 1.18%, 0.40% and 0.03%, while the PM2.5 (lag06), PM10 (lag06), NO_2_ (lag03), O_3_ (lag06) and CO (lag04) increased by 10 μg/m^3^, respectively.

### 2.2. The Impact of Health on Labor Mobility

In the 19th century, based on the law of population migration, E. G. Ravenstein first proposed the population movement theory [22]. In the 1960s, E. S. Lee (1966) [23] further perfected R. Herberle’s push–pull theory and argued that population migration could be attributed to the joint action of push and pull forces. Poor living conditions and various reasons push people away from their hometown, and good expectations pull people to new places. At the level of occupation, Thomas et al. (2006) [24] studied an intervention experiment in Indonesia, and the results showed that the improved health status significantly increased the probability of male rural residents participating in migrant work. Adhvaryu and Nyshadham (2017) [25] further suggested that most people with long-term illness experienced a shift from agricultural labor to enterprise labor, and sick people are more productive in business than on farms, and confirmed that disease is a push force for labor mobility. However, other scholars have opposite views. Jing et al. (2018) [26] found that health status had little impact on rural migrants’ choice of self-employment and employment.

In addition, based on the population migration theory, other scholars have explored the impact of health on labor mobility from the perspective of the Latino health paradox. The health paradox of Latin immigrants can be described as immigrants with lower socioeconomic status and fewer medical resources having better health outcomes than the local residents [27]. Many scholars generally believe that individual health status will significantly affect labor migration or mobility decisions, and have proposed the health depletion effect, the health migration effect [28,29] and the salmon bias effect [30]. The health migration effect suggests that workers with a good health status are more likely to leave home, and the salmon bias effect suggests that workers in poorer health status are more likely to return home. 

However, scholars do not fully support the viewpoints above. Indeed, when the labor supply is subjected to a health shock such as an epidemic, there is a temporary or permanent reduction in the labor supply due to the direct and indirect effects of illness and death. At the same time, the fear of being infected by others can lead to a decline in labor participation, and the closure of employment places [31]. If labor suffers a health shock such as COVID-19, this is likely to reduce the mobility inflow rate of different regions [32].

### 2.3. The Impact of Environmental Pollution on Labor Mobility

Since the early 1990s, scholars have been increasingly concerned about the relationship between environmental pollution and labor mobility. Among them, based on the dual economic structure framework, Harris and Todaro (1970) [33] proposed the classical Harris–Todaro model (“H–T model”), and discussed the relationship between labor transfer and environmental pollution. Based on the H–T model, some scholars have studied the relationship between the pollution and labor mobility from the industry sector perspective [34] and have confirmed that pollution in the industrial sector will affect the marginal income of the labor transfer, and further affect the labor mobility scale between urban and rural regions. Several scholars have studied the relationship between pollution and labor mobility from the population migration and the spatial mobility perspective [35] and have argued that a better quality of environment, including solid waste, the air, water, noise pollution and other factors, is the main factor in the interregional migration of individuals. That is different from the traditional view that economic factors are the dominant contributor to labor mobility.

Focusing on China’s labor mobility problem, most scholars have explored the influence of environment on labor mobility at a geo-space level. Their conclusions have shown that the natural environment has gradually become an attractive factor for labor immigrants [36], and air pollution exerts an important influence on labor mobility, especially for male people of a younger age with higher education, high skills or high-income [6]. However, they used air pollution or environmental pollution variables, such as haze and exhaust emissions, to explain the main impacts on labor mobility, and ignored the impact mechanism of the health shocks caused by air pollution on labor mobility. For example, using micro-survey samples in the Beijing-Tianjin-Hebei region, Lu et al. (2018) [7] argued that haze pollution has caused the technical talent loss in the Beijing-Tianjin-Hebei region. Few scholars have paid attention to the impact of environmental pollution on labor mobility between industries. The impact of environmental pollution on the employment scale shows an inverted U-shaped relationship, and the degree of environmental pollution also has an obvious impact on the employment structure of the industry [37]. 

**Figure 3:**
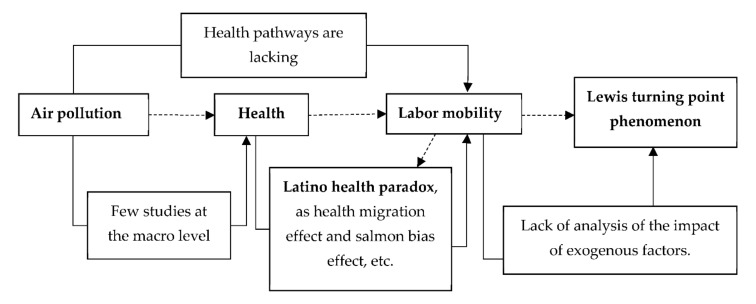
The theoretical framework.

To sum up, there are many studies on the relationship between air pollution and the health, the health and labor mobility, or air pollution and labor mobility, respectively. However, there are few studies on the relationship between air pollution, the health and labor mobility simultaneously. Meanwhile, studies on the relationship between air pollution and health lack the health-risk-generation perspective at the macro level, and the studies on the relationship between health and labor mobility do not further explain the health paradox of Latino immigrants due to the influence of air pollution. Finally, most studies on labor mobility involving the Lewis turning point are from the perspective of marginal productivity changes [38], and do not consider the impact of exogenous factors. Therefore, this article will focus on the relationship between air pollution, health and labor mobility at a macro level. The theoretical framework is shown in Figure 3.

### 2.4. Research Hypothesis

Indeed, China’s labor mobility index has declined, which is especially obvious in the labor force fleeing Beijing, Shanghai and Guangzhou, and labor shortages have restricted the high-quality development of the eastern region. However, areas such as eastern China and north of the Huai River are heavily polluted [11]. According to the reference above, there is a significant functional relationship between air pollution, health shocks and labor mobility. Therefore, we propose hypothesis H1 and H2:

**Hypothesis** **1** **(H1).** *Air pollution restricts labor mobility across provinces, and health shocks play a mediating role in that process.*

**Hypothesis** **2** **(H2).** *The impact of the health shocks of air pollution on labor mobility varies significantly in different regions. The severely polluted areas in eastern China and north of the Huai River, etc., perform more prominently.*

**Figure 4:**
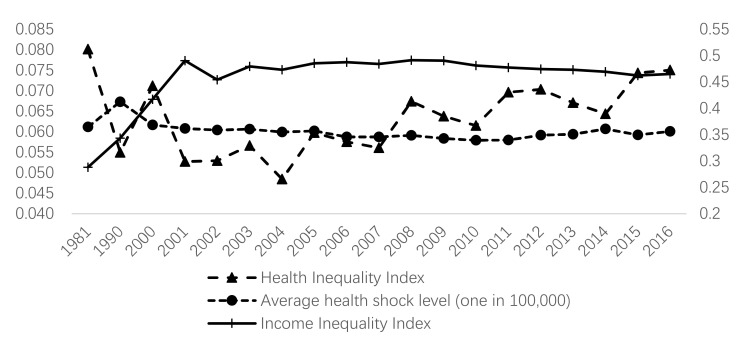
The annual development trend of residents’ income and health in China. Note: We used the geometric mean of the mortality rates in each province to represent the average health level, and adopted the annual income Gini coefficient to represent the income inequality index, and calculated the health inequality index in the sample using the Gini coefficient method.

Furthermore, as mentioned above, China’s labor mobility index and the annual average inflow of floating population fluctuated upward before 2010 and then downward. Since the reform and opening up, the continuous advancement of industrial priority and the eastern priority development strategy in China have not only accelerated the development of industrialization and urbanization, but also widened the income gap between different industries and regions, and created large-scale market-oriented spontaneous labor mobility, and the cross-regional scale of labor mobility is growing continuously. The nature of labor mobility is mainly affected by the inequality of development between regions and income equality. Subsequently, although income inequality has been slowly decreasing, the income gap is still obvious, and the average level of health shocks has changed only modestly after 2010, and health inequality between regions has further increased (Figure 4), while the labor mobility index has dropped significantly. Generally, we can conclude that the health shocks of air pollution on labor mobility have time differences, which mainly originated from the income inequality and air pollution [39]. Therefore, we propose hypothesis H3:

**Hypothesis** **3** **(H3).** *The health shocks of air pollution that hinder the inflow of labor mobility across provinces became more significant after 2010, indicating a threshold effect, and the threshold effect mainly originates from income and air pollution.*

## 3. Materials and Methods

### 3.1. The Econometric Model

#### Entropy Method

Firstly, we analyze whether the interaction between the air pollution index and health factors has a significant impact on labor mobility, and the benchmark model is as follows:

(1) $$l_{it}=\rho_{0}+\rho_{1}pol_{it}+\rho_{2}d_{it}+\rho_{3}pol_{it}*d_{it}+B*contr+\omega_{i}+\delta_{t}+\varepsilon_{it}$$

(2) $$l_{it}=\beta_{0}+\beta_{1}h_{it}+B_{0}*contr+\omega_{i}+\delta_{t}+\varepsilon_{it}$$

In Equations (1) and (2), *i*, *t* refers to the province and the year, respectively. The value *l* represents the labor mobility index, pol represents the composite index of air pollution, *d* denotes the health shock index, *pol*∗*d* is the interaction term, *h* represents the coupling index of air pollution and the health shock and *ω*, *δ* and *ε* refer to the individual effect, the time effect and the random effect, respectively. *B* and *B*_0_ represent the coefficient parameter vector of the control variables, *ρ*_0_ and *β*_0_ are the constants, *ρ*_1_, *ρ*_2_, *ρ*_3_ and *β*_1_ represent the coefficient parameters, and contr represents the control variables.

Secondly, we analyze the mediating effect of health shock in the impact of the air pollution on labor mobility, and model as follows:

(3) $$l_{it}=\alpha_{0}+\alpha_{1}pol_{it}+B_{1}*contr+\omega_{i}+\delta_{t}+\varepsilon_{it}$$

(4) $$d_{it}=\gamma_{0}+\gamma_{1}pol_{it}+B_{2}*contr1+\omega_{i}+\delta_{t}+\varepsilon_{it}$$

(5) $$l_{it}=\lambda_{0}+\lambda_{1}pol_{it}+\lambda_{2}d_{it}+B_{3}*contr+\omega_{i}+\delta_{t}+\varepsilon_{it}$$

In Equations (3)–(5), *B*_1_, *B*_2_, *B*_3_, *α*_1_, *β*_1_, *γ*_1_, *λ*_1_ and *λ*_2_ represent the coefficient parameters, respectively. *α*_0_, *γ*_0_, and *λ*_0_ are the constants. *contr*1 represents the control variables. When *α*_1_ in Equation (3) is significant, it indicates that air pollution has an overall influence on labor mobility. Subsequently, Equations (4) and (5) test whether the *γ*_1_, *λ*_1_ and *λ*_2_ are significant, indicating that air pollution affects labor mobility partly by a health shock; if *γ*_1_ and *λ*_2_ are significant on the test, while *λ*_1_ is not, the effect of air pollution on labor mobility is entirely due to a health shock. Finally, if *γ*_1_ or *λ*_2_ show only one or none of the tests to be significant, a Sobel or bootstrap test is required to further determine the mediating effect.

Lastly, we adopt the threshold effect model, to further explore to what level the health shock of air pollution influences labor mobility, and the model as follows:

(6) $$l_{it}=\mu_{0}+\mu \times M\times I(\cdot )+\omega_{i}+\varepsilon_{it}$$

In Equation (6), *μ*_0_ refers to the constant, *μ* refers to the coefficient and *M* is the vector set of explanatory variables in Equation (1). *I*(∙) represents the indicative functions of each threshold condition. The time effect is not controlled here, as the threshold condition contains a time variable.

### 3.2. The Variable Measurement

#### 3.2.1. The Dependent Variables

Referring to Lin and Wang (2006) [40], the population changes caused by the natural population growth were excluded from the total population changes in each region, and the working population aged 15–64 was retained to obtain the net population change scale as the net inflow of labor mobility. Indeed, according to China’s Sixth National Census in 2010, 90% of the floating population are of working age [3]. Thus, the net inflow of population multiplied by 90% is an estimate of the net inflow of labor mobility, and can be regarded as the labor mobility index *l*, and the index *l_it_* in region *i* from time *t* to *t* + 1 can be calculated as:

(7) $$l_{it}=\frac{(p_{it+1}-p_{it}\times (1+n_{it}))\times 90\%}{(p_{it}{}_{-1}+p_{it})/2}$$

In Equations (7), *p* refers to the year-end population in one region. *l_it_* >0 denotes the net inflow and *l_it_* <0 denotes the net outflow.

#### 3.2.2. The Explanatory Variables

The index of health shock effect (d) and the index of air pollution (pol), measured by Zhang and Wang (2020) [3] represent the health shock level (referring to the combined impact level including the air pollution impact and the impact of other factors) and the air pollution level, respectively. h is the coupling measure index of air pollution and health shocks, directly representing or specifically referring to the health shock index of air pollution. 

To overcome the different dimensional effects, the multicollinearity and the subjective empowerment arbitrariness of one complex system with more indicators, and avoid the information loss caused by the individual effect and the time effect, we adopt the principal component-entropy weight method. This article analyzes the time and the space value of the selected variables after standardization, and the relative risk values of the two dimensions are, respectively, *q* and *r* in the final measure. Meanwhile, in order to better reflect the individual differences among the different samples, the composite index *g* containing the time and space dimensions is:

(8) $$g=e^{q}*e^{r}=e^{\left(q+r\right)}$$

Based on the above, to calculate the *pol*, *d* and *h* value, the specific measurement steps are as follows:

Firstly, the samples were extracted after standardization, and the principal component dimension reduction analysis was performed to extract the principal components for air pollution and health shock subsystems.

Secondly, the standardized score of the extracted principal components is calculated in each subsystem. Then, using the entropy weight method, the entropy information is extracted from samples, and the weight of each index in the time and the space dimensions is determined.

Finally, using the obtained weight, the relative index values *q*(*pol*), *r*(*pol*), *q*(*d*), *r*(*d*), *q*(*h*) and *r*(*h*) of the spatial and temporal dimensions were calculated, and then the air pollution index (*pol*), the health shock index (*d*), and the coupling index *h* of the air pollution system and the health shock system is calculated as follows:

(9) $$pol=e^{\left(q(pol)+r(pol)\right)}$$

(10) $$d=e^{\left(q(d)+r(d)\right)}$$

(11) $$h=e^{\left(q(h)+r(h)\right)}$$

#### 3.2.3. The Control Variables

The set (contr) of control variables in Equations (1)–(3) and (5) includes:

(1) The real per capita income (gp). The gp is calculated by the proportion of the real per capita GDP on the commodity housing price. Some scholars believe that air pollution is one of the important factors affecting the housing price, and the regional housing price is also one of the important factors influencing labor mobility [41], and the regional housing price factors should be excluded from the regional per capita income. After excluding the regional housing price factors, the region with the higher per capita real income level will be more likely to attract labor inflow [42];
(2) The regional openness (iemport). The iemport is calculated by the proportion of the total imports and exports on GDP, and the region with higher openness will have more job opportunities to attract labor inflow [42];
(3) The regional education level (pere). Education has an important impact on labor migration [43]. The pere is calculated by the weighted education level per capita, and the region with the higher education level is more likely to be a developed region, and to attract labor inflow (the specific calculation formula of per capita education level is as follows: the average number of years of education for the labor force =the proportion of illiterate and semi-illiterate in the employed population *1.5+ the proportion of the employed population receiving primary education);
(4) The industrial structure (thr). Industrial structure is also an important variable affecting labor mobility [44]. The thr is calculated by the proportion of the tertiary industry on GDP. The more developed the tertiary industry is, the more likely it is to attract labor inflow;
(5) The average daily precipitation (rain). The precipitation is also an important factor affecting labor mobility [44]. The rain is calculated by the ratio of annual precipitation to the number of days in the corresponding year. The more the average daily precipitation, the less sunshine there will be, and the more likely it is not conducive to labor inflow;
(6) The per capita road area (perroad). The perroad is calculated by the road area divided by the number of people at the end of the year. The higher the per capita road area, the better urban transportation and infrastructure in one region, the more likely it is to attract labor inflow [45].

The set (contr1) of control variables in Equation (4) includes:

(1) The per capita GDP (pergdp). The pergdp represents the regional economic development level. It is generally believed that the higher the economic level is, the better it is able to resist health shocks [46];
(2) The regional openness (iemport). The higher openness in one region, the more likely it is to acquire new technologies and improve health [47]. However, other scholars have confirmed that higher pollution in one region is always correlated with higher openness [48], which will be worse for resisting health shocks;
(3) The per capita education level (pere). The education level is an important variable affecting the health level [30]. In a region with higher per capita education level, people’s health awareness will be stronger than in others, and the better they will be able to resist health shocks;
(4) The average temperature (temp). The temp is substituted by the average temperature of major cities in each province, and it is an important exogenous factor affecting people’s health or disease [14];
(5) The number of health technicians per 1000 population (tec). The medical level is also an important variable affecting health level [30]. The higher medical care level in a region, the better it is able to resist health shocks.

### 3.3. The Sources of Materials

Without special statements, the data used in this article are from provincial statistical yearbooks of China, China Stock Market and Accounting Research Database (CSMAR), China Economic Network Statistical Database, China Statistical Yearbook, China Health Statistics Yearbook, China Environmental Statistics Yearbook and the meteorological monitoring data of Columbia University, etc. In this paper, the sample time ranges from 2007 to 2015; most health indicators’ statistic values start from 2007, and this can avoid the exogenous interference of related policies before 2016, as China implemented the Healthy China 2030 Development Strategy in 2016. We also establish panel data from 30 provinces (excluding Hong Kong, Macau, Tibet and Taiwan) from 2008 to 2015, and adjust some variables, such as GDP per capita imports and exports, to avoid influence from inflation. Table 1 shows the descriptive statistics of the variables. 

## 4. Results

### 4.1. Characterization of Main Variables

**Figure 5:**
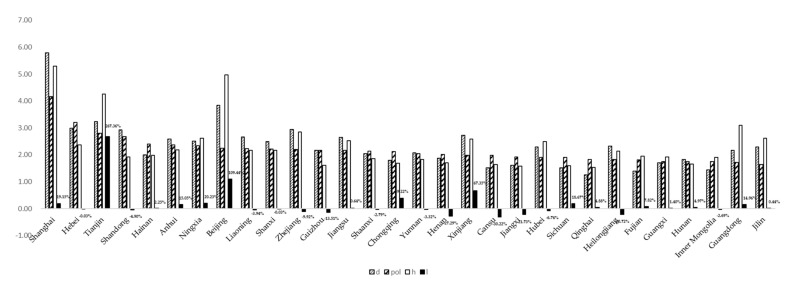
The changes of key variables in 30 provinces in recent years (average value in recent three years). Note: Because the labor mobility index is generally low, numerical labels have been added to show the results clearly; others have not been added.

Based on the measurement results of the above methods, from the annual average of the past three years (see Figure 5), the air pollution index (*pol*), health shock index (*d*) and the health shocks composite index of air pollution (*h*) vary greatly among provinces, and the index of labor mobility (*l*) is generally low.

**Figure 6:**
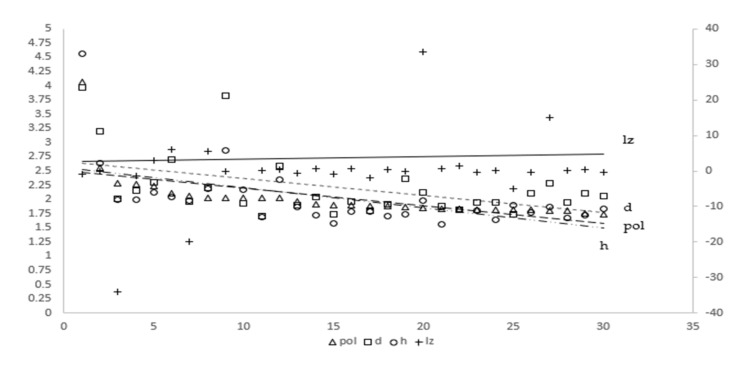
The annual average value and trend of several index measurement results in various provinces. Note: The serial numbers 1–30 on the x-axis represent Shanghai, Tianjin, Hebei, Jiangsu, Ningbo, Guangdong, Shanxi, Liaoning, Beijing, Shandong, Guizhou, Zhejiang, Chongqing, Hainan, Sichuan, Yunnan, Henan, Qinghai, Fujian, Guangzhou, Gansu, Anhui, Shaanxi, Inner Mongolia, Hunan, Hubei, Xinjiang, Jiangxi, Heilongjiang and Jilin.

Furthermore, the annual average growth rate of the labor mobility index (*l_z_*, *l_zt_* = (*l_t_*/*l*_*t*−1_) − 1), the annual average value of air pollution index (*pol*), the annual average value of the health shock index (*d*) and the annual average value of the health shocks composite index of air pollution (*h*) of each province are shown in Figure 6. We can see that the *l_z_* line shows a positive tendency, and other three lines are showing a negative trend, indicating that the growth in the health shocks of air pollution may be closely related to the decline of the net inflow rate of labor mobility. We will confirm this in the next section.

### 4.2. The Baseline Results

A fixed or random model is adopted according to the Hausman test. A dynamic system GMM model was adopted to estimate the first-order hysteresis effect and to reduce the endogenous problem, such as the potential autocorrelation effect of the net inflow rate of labor mobility, and the potential impact of the agglomeration effect formed by labor mobility on environmental pollution [49], and Table 2 shows the estimation results.

In column (1), the coefficient of the air pollution index is significantly negative at the 5% confidence level, indicating that air pollution will reduce the net inflow of labor mobility, and the negative impact is further confirmed in column (4). As shown in column (4), for each 1% increase in the air pollution index, the net inflow of labor mobility will drop by 47.6%, significant at a 1% confidence level. Using a sample of 285 cities in China, Zhang (2019) [50] suggested that the number of employed workers will be reduced 33.45% with the SO_2_ emissions increasing 1%, and our results further confirm the negative impact of air pollution on labor mobility. Using the comprehensive calculated air pollution, not a single pollutant, as the air pollution index, the negative impact in our article is larger than others. In column (2), the coefficient of the interaction term between air pollution and health shocks is significantly negative at the 5% confidence level. This implies that the net inflow of labor mobility will be reduced by the interaction of air pollution and health shocks, and the endogenous problem underestimates the negative impact shown in column (5). In column (5), when controlling other conditions, for each 1% increase in the interaction item of air pollution and health shocks, the net labor inflow of labor mobility will drop by 15.7%, significant at the 1% confidence level. In column (3), the coefficient of the health shock index of air pollution is significantly negative at the 5% confidence level, indicating that the net inflow of labor mobility will be decreased 24.9%, with a 1% increase in the health shock index of air pollution. In column (6), a dynamic system GMM model is adopted that confirms the negative impact of the health shock index of air pollution on the net inflow of labor mobility. Therefore, we can see that the health shocks of air pollution are a significant hindrance to the net inflow of labor mobility, and confirm the related hypothesis in H1. 

### 4.3. The Mediation Effect Results

Based on Equations (3) to (5), the mechanism effect results are shown in Table 3. The variable pol has a significant negative impact on the net inflow of labor mobility at the 5% confidence level in column (7). In column (8), the coefficient of the air pollution index is significantly positive at the 10% confidence level, indicating that there is a negative impact of the air pollution index on health shocks, and health shocks will be increased 16.5% with an air pollution index increase of 1%. The coefficient of health shocks is significantly negative at the 1% confidence level in column (9). However, the coefficient of the air pollution index is not significant in column (9), which means that it exerted a full mediation effect. Therefore, it can be concluded that the impact of air pollution on the labor inflow is all caused by health shocks, and that rectifying health shocks is also an important factor influencing the decrease in the labor supply and migrating individuals with different health levels under different air pollution conditions.

In order to further ensure the reliability of mediating effect results, the Sobel test is used in this paper. In accordance with Sobel statistics [51], the Sobel values and the *p* values were directly calculated using the coefficients of each major effect, and the Sobel 95% confidence interval was [1.616, 1.738], with a significant value of −1.67 < −0.97 at the 5% confidence level, confirming the mediating effect. As mentioned above, the health shocks of air pollution restrict the net inflow of labor mobility, and the hypothesis H1 has been verified.

### 4.4. The Robustness Test

Based on the current study, we adopt two main ways to test the robustness of the results above, and Table 4 shows the result. Firstly, we use one lagging term based on the main explanatory variables, and the results are displayed in column (10) to column (13). We can see that the coefficients of the variables pol*d and h are significantly negative at the 10% (or less) confidence level, and confirm the stability of the results above. Secondly, according to the idea of hedonic price, the housing price can measure the labor inflow in one region [52]. However, the housing price does not reflect the real market demand in China influenced by the housing market bubble. Therefore, Xi and Liang (2015) [53] used the idea of choosing a housing sales area as a proxy variable to analyze the effect of environmental migration. Meanwhile, to weaken the scale effect of the provincial population and the influence of natural growth, again referring to Zhang and Wang (2020) [3], we use the natural logarithm of the commodity housing sales area per (lniv6) as the proxy variable of the labor mobility index, and the results are shown in column (14) to column (17). We can find that most coefficients of the variables h, pol and d are significantly negative at the 10% (or less) confidence level, indicating that the conclusion above is robust.

In addition, we use the lagged term of the air pollution index and the health shock index to test the medication effect, and Table 5 shows the results. In Table 5, we can see that the impact of air pollution on the net inflow of labor mobility is mainly caused by the mediating effect of health shocks. Thus, the reliability of the mediating effect above is rectified (MacKinnon et al. (1995) [54] set 0.97 as the boundary value at the 5% significance level for testing distribution).

### 4.5. Results of Regional Conditions

Furthermore, from the regional perspective, we analyze the impact of the health shocks of air pollution (h) on the index of labor mobility (l). Since the Seventh Five-Year Plan, China has been strategically divided into four economic development zones in the east, center, west and northeast. Now, these have been redivided into five major economic belts of “two horizontal and three vertical.” In order to find the regional differentiates of the samples, we choose the eastern, central and western economic belts and the Yangtze River economic belt as the heterogenous conditions. Scholars believe that the difference in air pollution on the Huai River between the north and south is large, and the health-damaging effects are also accordingly different [6,11]. Therefore, it is necessary to analyze the regional differentiates according to the boundary of the Huai River. The results are shown in Table 6.

The health shocks of air pollution in the east have significantly hindered the index of labor mobility. When other conditions remain unchanged, for every unit of the health shocks composite index of air pollution, the net inflow rate of labor mobility drops significantly by 33.1%, while the impact in the central and western regions is not significant. This result indicates that the labor force in the eastern region has fled, facing severe health shocks from air pollution. According to the sample, compared with 2007, in 2015, the net inflow rate of labor mobility in the eastern region declined by 86.35%, while that in the central and western regions increased by 85.24% and 196.44%, correspondently.

The impact of the health shocks of air pollution on labor mobility in the Yangtze River economic belt is significantly negative. When other conditions remain unchanged, for each unit increase in the health shocks of air pollution, the net inflow rate of labor mobility drops significantly by 34.6%. This is in line with the fact that there were 12,158 chemical enterprises above the designated size in the Yangtze River economic belt in 2016, accounting for 46% of those in China, with 41% heavy industry among them, forming a “chemical industry surrounding the river” with high pollution emissions. This result implies that in the process of promoting high-quality development, the negative impact of health shocks from air pollution on labor mobility cannot be ignored in the Yangtze River economic belt. Indeed, the rectification of chemical enterprises in the Yangtze River economic belt also took place after 2015, and this effectively avoided the exogenous effects of labor “flight.”

The impact of the health shocks of air pollution on labor mobility in the northern part of the Huai River is significantly negative, while not being significant in the southern part. When other conditions remain unchanged, for each unit increase in the health shocks of air pollution, the net inflow rate of labor mobility in the northern Huai River drops significantly by 31.8 %. Because the heating supply has increased more than air pollution only in the northern part of the Huai River, the impact of health shocks of air pollution on labor mobility in north is accordingly obvious [6]. Therefore, the hypothesis H2 has been verified.

### 4.6. The Threshold Effect Results

According to the current study, the health shocks caused by air pollution changed greatly after 2010 [39]. We chose 2010 as the time threshold variable, to confirm the impact of the health shock of air pollution on the Lewis inflection point theory. We also chose the health shocks of air pollution as a threshold variable to explore the level of the health shocks of air pollution’s influence on labor mobility, and the results are shown in Table 6. In Table 6, whether time or the health shocks of air pollution are the threshold variable, the null hypothesis cannot be accepted without a threshold in the single threshold situation, and the alternative hypothesis test with two or three thresholds fails, indicating that there is one threshold. Table 7 and Table 8 show the estimated results with a single threshold variable, and Table 9 shows the impact effect estimation of threshold points.

In Table 8, the time threshold value is 2010 at the 5% confidence level. In column (27), the coefficient of the time threshold before 2010 is not significant, and the coefficient of the time threshold after 2010 is significantly negative at the 1% confidence level, indicating that the negative effect of the health shocks of air pollution on labor mobility is not significantly obvious before 2010, and the negative effect is obvious after 2010. This result shows that the health shocks of air pollution in China have intensified since 2010.

**Figure 7:**
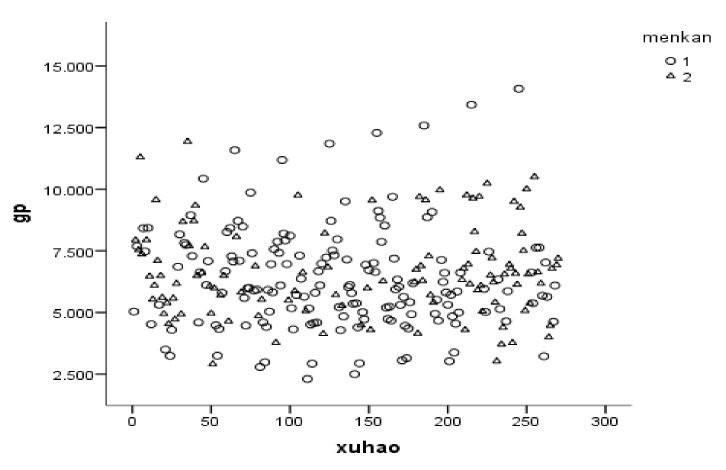
The sample distribution of the real income level under the two risk types of the health shocks of air pollution. Note: Level 1 represents the health shocks of the air pollution risk <1.9873, and level 2 represents ≥1.9873.

In Table 8, the threshold value of the health shocks of air pollution is 1.9873 at the 5% confidence level. In column (28), the coefficient of the variable h is −0.523 and −0.389 at the 1% confidence level, respectively. This indicates that the health shocks composite index of air pollution has one threshold effect on the net inflow of labor mobility, and the impact will decrease when the health shocks of air pollution cross the threshold value. In order to explore this interesting phenomenon, Figure 7 shows the sample distribution of real income level under the two risk types of the health shocks of air pollution (there are two reasons to choose the variable of real income. First, since the reform and opening up, one of the main purposes of labor mobility between urban and rural areas in China is to increase family income, and improve family economic conditions. Second, the persistence of severe air pollution in China has been accompanied with extensive economic growth over a long period, and this growth can be reflected in real income factors. Therefore, we chose the real income as the representative factor of the gravity effect for analysis.). The average per capita real income level in type one and two regions is 6.656 and 6.339, respectively, and the percentage of the value is more than 7.106 (the threshold value of 7.106 is obtained by the clustering per capita real income (gp) according to the K-means). The proportion obtained by contingency table analysis is 31.2% and 31.1%, and the standard deviation value is 1.860 and 2.106, respectively, among them. We can find that the average per capita real income, and its percentage, with a value of more than 7.106 in type two is larger than in type one, while the standard deviation value corresponding to type two is less than type one, indicating that the stable and higher real income plays a strong attracting role on the net inflow of labor mobility when the health shocks of air pollution crosses the threshold value, and the attracting effect partially offsets the repulsion effect of the health shocks of air pollution on the inflow of labor mobility.

**Figure 8:**
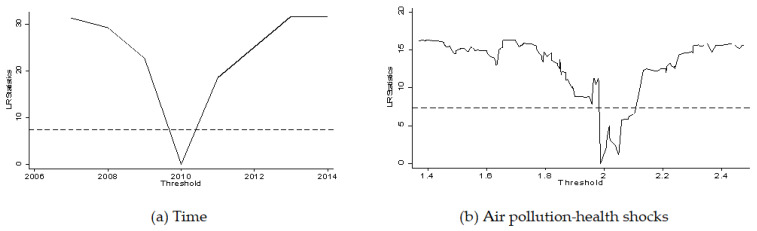
The LR statistics value trend estimated for the threshold variable.

In addition, Figure 8 shows the LR statistics value trend estimated for the threshold variable. We can find that the LR values in 4(a) and 4(b) are less than the critical value 7.35 at the 5% confidence level (the dashed line), further confirming the validity of the threshold effect (due to space limitations, other threshold LR test results are omitted). Therefore, hypothesis H3 has been verified, and labor mobility in China is currently simultaneously affected by income and the health shocks of air pollution.

## 5. Discussion

This article used the principal component entropy weighted method to calculate the air pollution composite index and health shock index. Most studies have chosen the AQI or the PM2.5, alternative variables of air pollution, to analyze the impact of air pollution on labor mobility [5,6], but one pollutant, such as in the PM2.5, cannot fully represent the total air pollution, and the calculation process of the AQI index does not eliminate the correlation between different pollutants. The principal component entropy weighted method can identify the most important information of air pollution and health shocks, and avoid the defect of the AQI index and the single air pollutant, to make the conclusion more reliable. 

Based on the air pollution composite index and the health shock index above, this article analyzes the impact of air pollution on labor mobility, and the results show that labor mobility is affected by the health shocks of air pollution. Similar results were found by Cropper (1981) [55] and Sun et al. (2019) [39]. However, most studies focused on the impact factors such as the housing price or environmental regulation [56,57] and ignored the impact of health shocks. Adopting the mediation effect model, our results further imply that the impact of air pollution on labor mobility is all caused by health shocks, and health shock is a very important factor of air pollution’s impact on labor mobility at the macro level. We can explain these results from two angles: firstly, although individuals have the motivation to invest in their health and human capital, they will display mobility behavior to avoid air pollution, and show more as a moderating effect to avoid health shock [58]. Secondly, when one region faces a serious health shock caused by environmental pollution, it will firstly exclude migrant labor in a poor health condition, and eventually extract the migrant population in a good health condition, resulting in the health level of the migrated population being higher than that of the locals, as described in the Latino health paradox. This phenomenon is similar to the health paradox of Latino immigrants with a health self-screening mechanism [59].

Using the threshold model, our results further show that the health shocks of air pollution on labor mobility across provinces became more significant after 2010, and we confirm the view that China’s Lewis turning point was 2010 [60]). However, compared with most current views [61], our results more strongly emphasize health shocks in China’s Lewis turning process and its avoidance effect, and argue that the health shocks of air pollution are also an important reason for the decline of labor mobility supply across provinces. It has an obvious difference with the impact of different wages caused by the urban–rural dual structure. Indeed, the impact of the health shocks of air pollution on labor mobility will increase the labor’s burden of medical expenses, and decrease the labor’s expected income and life expectancy [62]. In addition, when the labor anticipates that there may be a large health shock in one region with severe environmental pollution, the labor will reduce its mobility intention, and the regional labor inflow rate will decline.

Finally, based on the push–pull theory, we conclude that the attraction effect of regional stability and high real income will partially offset the repulsion effect of the health shocks of air pollution on labor mobility, when the health shock index of air pollution exceeds the threshold value of 1.9873, similar to the view of Li et al. (2020) [63]. However, most studies focus only on the negative impact of air pollution on labor mobility. Environmental pollution has null jointness. It is not only a byproduct of production, but also an important input of production [64], especially in developing countries or regions, and the regional economy can quickly achieve extensive growth with a corresponding improvement in pollution levels.

Compared with the empirical conclusions of previous studies, we found similar conclusions, with some differences. We confirm the negative impact of air pollution on labor mobility that Chen et al. found (2017) [5,6]. However, we find the impact of air pollution on labor mobility is all caused by health shocks, and this result is obviously different from most studies, such as that of Kahn and Mansur (2013) [56]. In addition, we find the threshold effect of time and health shocks of air pollution, and confirm China’s Lewis turning point was 2010, in a manner similar to Kwan et al. (2018) [60]. However, our study has several limitations. Firstly, the study period ranged from 2008 to 2015. For policy effect interference and data limitation, we did not extend the data to 2020 or 2021, and the statistical bias may underestimate the negative impact of air pollution on labor mobility. Second, the air pollution index and health shock index were calculated by the principal component entropy weight method, and do not consider the weight valued by related experts. This might have resulted in the underestimation of the mediation effect of health shocks. Thirdly, this article did not use the geographical method to display the spatial labor mobility and spatiotemporal impact of air pollution on labor mobility. Therefore, we may have to empirically analyze these in a further study.

## 6. Conclusions and Policy Implications

### 6.1. Conclusions

This paper anchors the perspective of health shocks, and discusses the relationship between air pollution, health shocks and labor mobility based on China’s macro panel samples. The main conclusions are as follows:

1. Air pollution can have a negative impact on the net inflow of labor mobility, air pollution will reduce the net inflow of labor mobility, the net inflow of labor mobility will be reduced by the coaction with air pollution and health shocks and the net inflow rate of labor mobility will decrease between 24.9% and 44.7% with each unit increase in the health shocks of air pollution. The robustness tests confirm the reliability of these conclusions;
2. The impact of air pollution on labor mobility is entirely caused by health shocks; health shocks are also an important factor influencing the decrease in labor mobility supply across provinces and the different health levels of the migrating individuals with respect to different air pollution;
3. The health shocks of air pollution have a single time threshold effect on labor mobility, and the health shocks of air pollution in China have intensified since 2010. This result confirms China’s Lewis turning point was 2010, and health shocks play an important role in China’s Lewis turning process and the avoidance effect, similar to the phenomenon of the Latino immigrants’ health paradox;
4. When the index of health shocks from air pollution exceeds the threshold value of 1.9873, the attraction effect of regional stability and higher real income will partially offset the repulsion effect of the health shocks of air pollution on labor mobility.

### 6.2. Policy Implications

Based on the conclusions above, the policy implications are as follows:

1. Increase the investment in air pollution control to reduce the risk of the health shocks caused by air pollution. Although tackling pollution will decrease the economic growth rate, the higher the health shocks of air pollution, the more they will hamper labor mobility. Therefore, a reasonable increase in investment in air pollution control can reduce the risk of health shocks, stabilize the labor supply and promote high-quality development.
2. Continuously increase the related public expenditure for labor mobility and health protection. This should provide more public expenditure on the floating population, especially. It should highlight the priority development of people’s health and pay special attention to environmental health education and training for the floating population. Additionally, improve the public expenditure on medicine and education, to improve the labor’s environmental and health awareness, and establish a health shock defense and guarantee mechanism for the floating population to facilitate the rational labor mobility.
3. Implement and strengthen the evaluation of attracting factors, and establish a mechanism to guide labor mobility and work assessment. These results show that the attracting factors, such as stable and higher real income, can partially offset the negative impact of the health shocks of air pollution on labor mobility, and it is necessary for local government to establish an income improving scheme, and especially to control the stability of the housing price and enlarge the supply of the public rental housing to attract more people with a higher education level.

## Figures and Tables

**Table 1:** The descriptive statistics of the variables.

Variable	Description	Unit	Mean	Std.Dev.	Min	Max
l	The index of labor mobility	%	0.279	1.163	−5.360	5.077
h	The health shocks composite index of air pollution		2.012	0.797	1.085	7.389
pol	Air pollution index		2.029	0.563	1.164	4.552
d	Health shock index		2.206	0.715	1.284	6.185
pergdp	Per capita GDP	yuan/person	33,514.940	19,462.150	6915.000	106,184.200
gp	Per capita real income level	m^2^/person	6.467	2.013	2.300	14.075
iemport	The ratio of total imports and exports to GDP	%	0.146	0.224	0.000	1.101
pere	Average years of education	year/person	10.864	1.128	8.267	14.610
thr	Ratio of output value of tertiary industry to GDP	%	41.342	8.823	28.600	79.653
rain	Average daily precipitation	100 million m^3^	4.954	3.467	0.151	13.965
perroad	Road area per capita	m^2^/person	13.543	4.289	4.040	25.820
temper	Average temperature	Celsius	14.474	5.045	4.300	25.333
tec	Number of health technicians per 1000 population		5.094	1.912	2.140	15.460

Note: The sample size is 270.

**Table 2:** The baseline main results of OLS and GMM models.

Var.	(1)	(2)	(3)	(4)	(5)	(6)
	OLS(FE)	OLS(RE)	OLS(FE)	GMM	GMM	GMM
L.l				0.338 **	0.379 **	0.287 **
				(2.23)	(2.46)	(2.25)
pol	−0.308 **	−0.100		−0.476 ***	−0.207	
	(−2.50)	(−0.92)		(−3.13)	(−1.49)	
d		−0.358*			−0.165	
		(−1.75)			(−0.76)	
pol∗d		−0.172 **			−0.157 ***	
		(−2.19)			(−2.87)	
h			−0.249 **			−0.429 ***
			(−2.58)			(−4.67)
lngp	1.415 **	0.446	1.148 **	0.334 *	0.309	0.496 ***
	(2.46)	(1.60)	(2.10)	(1.87)	(1.15)	(2.95)
iemport	3.811 ***	1.539 ***	2.844 ***	1.068 **	1.166 **	0.938 **
	(4.36)	(3.36)	(3.81)	(2.46)	(2.02)	(2.31)
lnpere	4.529 **	3.714 ***	3.486 **	0.546	0.752	1.016
	(2.72)	(2.97)	(2.27)	(0.66)	(0.95)	(1.11)
lnthr	0.193	0.982	0.038	0.496	0.549	0.973 *
	(0.22)	(1.16)	(0.04)	(1.21)	(1.09)	(1.80)
lnrain	−0.253	−0.377 ***	−0.297	−0.275 ***	−0.281 **	−0.289 **
	(−0.92)	(−2.62)	(−1.09)	(−2.58)	(−2.12)	(−2.53)
perroad	0.134 *	0.000	0.132 *	−0.013	−0.016	−0.020
	(1.98)	(0.00)	(2.00)	(−0.85)	(−1.00)	(−0.96)
Constant	−14.601 **	−11.485 ***	−11.040 *	−2.270	−3.023	−5.413 **
	(−2.43)	(−2.86)	(−2.04)	(−0.80)	(−1.19)	(−2.06)
Time effect	Y	Y	Y	Y	Y	Y
Observations	270	270	270	240	240	240
Number of provinces	30	30	30	30	30	30
R2	0.172	0.5574	0.183			
F/Wald	3.490	548.56	2.847	115.33	386.24	137.56
AR(1)				0.047	0.041	0.042
AR(2)				0.133	0.147	0.176
Hansen				0.996	1.000	0.987

**Note:** *, **, *** refers to significance at the level of 10%, 5% and 1%, respectively. The number in parentheses is t value. The Wald values in the GMM model are shown by F/Wald, and the others shown by F values. The same below.

**Table 3:** The estimation results of the mediation effect.

Var.	(7)	(8)	(9)
	Labor Mobility	Health Shocks	Labor Mobility
pol	−0.308 **	0.165 *	−0.139
	(−2.50)	(1.97)	(−1.11)
d			−0.546 ***
			(−3.16)
lngp	1.415 **		0.872 *
	(2.46)		(1.87)
iemport	3.811 ***	−2.052 ***	1.956 **
	(4.36)	(−2.92)	(2.34)
lnpere	4.529 **	−2.472	−1.836
	(2.72)	(−1.50)	(−1.43)
lnthr	0.193		−0.436
	(0.22)		(−0.77)
lnrain	−0.253		−0.157
	(−0.92)		(−0.76)
perroad	0.134 *		0.081
	(1.98)		(1.66)
lnpergdp		−2.406 *	
		(−1.78)	
lntemper		0.155	
		(0.32)	
lntec		−1.236 ***	
		(−3.52)	
Constant	−14.601 **	32.794 **	4.974
	(−2.43)	(2.52)	(1.24)
Number of observations	270	270	270
Adjusted R2	0.172	0.492	0.234
F	3.490 ***	9.484 ***	4.991 ***
Individual effect	Y	Y	Y
Time effect	Y	Y	N

Note: *, **, *** refers to significance at the level of 10%, 5% and 1%, respectively. The number in parentheses is t value. The Wald values in the GMM model are shown by F/Wald, and the others shown by F values. The same below.

**Table 4:** The robustness test results of the baseline model.

Var.	(10)	(11)	(12)	(13)	(14)	(15)	(16)	(17)
	Test Model of Lag Term				Test Model of Tool Variable (lniv6)			
	OLS(RE)	GMM	OLS(FE)	GMM	OLS(FE)	GMM	OLS(RE)	GMM
L.l		0.160		0.218				
		(1.24)		(1.39)				
L.lniv6						0.679 ***		0.705 ***
						(6.03)		(5.63)
L.pol	−0.147	−0.109						
	(−0.83)	(−0.79)						
L.d	−0.454 *	−0.295 *						
	(−1.80)	(−1.66)						
L.(pol∗d)	−0.278 **	−0.338 ***						
	(−2.06)	(−3.28)						
L.h			−0.428 *	−0.378 **				
			(−2.02)	(−2.00)				
pol					0.038	−0.129 *		
					(0.84)	(−1.74)		
d					−0.163 ***	−0.069		
					(−3.22)	(−1.36)		
pol∗d					−0.051 *	0.052 **		
					(−1.79)	(2.32)		
h							−0.083 ***	−0.146 ***
							(−2.72)	(−2.69)
Control variables	Y	Y	Y	Y	Y	Y	Y	Y
Time effect	Y	Y	Y	Y	Y	Y	Y	Y
Observations	240	240	240	240	270	240	270	240
Number of daima	30	30	30	30	30	30	30	30
r2_a	0.5272		0.204		0.509		0.3546	
F/Wald	509.95	167.75	3.066	68.80	38.98	382.38	141.13	297.09
AR(1)		0.045		0.048		0.002		0.001
AR(2)		0.192		0.090		0.062		0.367
Hansen		0.441		0.160		0.999		0.891

Note: *, **, *** refers to significance at the level of 10%, 5% and 1%, respectively.

**Table 5:** The robustness test results of the mediation model.

Var.	(18)	(19)	(20)
	Labor Mobility	Health Shocks ^1^	Labor Mobility
L.pol	−0.363 *		−0.226
	(−1.77)		(−1.36)
pol		0.165 *	
		(1.97)	
L.d			−0.684 ***
			(−3.91)
Adjust the R^2^	0.157	0.492	0.276
F	3.774	9.484	6.690
Individual fixation effect	Y	Y	Y
Time fixation effect	Y	Y	Y
Control variables	Y	Y	Y

Note: *, *** refers to significance at the level of 10% and 1%, respectively. ^1^ According to the mediation model in Equation (2), the pollution index and the health-shocks index should both be regressed at the same time with a lag, but there are still one-to-one correspondences in the same period. Therefore, the actual operation does not use a lag period.

**Table 6:** Estimated results of heterogeneous conditions.

Var.	(21)	(22)	(23)	(24)	(25)	(26)
	The Eastern Region	The Central Area	The Western Region	Yangtze River Economic Belt	North of Huai River	South of Huai River
h	−0.331 **	0.072	0.055	−0.346 ***	−0.318 *	−0.218
	(−2.61)	(0.54)	(0.59)	(−5.43)	(−1.89)	(−1.33)
R-squared	0.718			0.363		
Control variables	Y	Y	Y	Y	Y	Y
Individual fixed effect	Y	N	N	Y	N	N
Time fixed effect	Y	Y	N	N	N	N
Observations	99	72	99	99	135	135

Note: *, **, *** refers to significance at the level of 10%, 5% and 1%, respectively.

**Table 7:** Indicators of driving forces of urban resilience.

Time Threshold Test	RSS	MSE	Fstat	Prob	Crit10	Crit5	Crit1
Single ***	94.515	0.362	34.140	0.000	10.361	12.291	17.772
Double	94.052	0.360	1.280	0.713	7.364	11.480	18.015
Triple	93.761	0.359	0.810	0.673	3.383	4.187	6.473
The threshold test of air pollution-health shocks	RSS	MSE	Fstat	Prob	Crit10	Crit5	Crit1
Single **	101.840	0.390	12.910	0.037	9.811	11.732	16.100
Double	99.492	0.381	6.160	0.360	9.479	11.367	14.554
Triple	90.635	0.347	25.510	0.100	25.442	37.189	60.642

Note: **, *** refers to significance at the level of 5% and 1%, respectively.

**Table 8:** The estimation of single threshold point.

Th-1	Threshold	Lower	Upper
Time threshold	2010	2009	2011
The threshold of air pollution-health shocks	1.9873	1.9577	2.001

**Table 9:** The impact effect estimation of threshold points.

Var.	(27)	(28)
	Time Threshold	The Threshold of Air Pollution-Health Shocks
0b._cat#c.h	0.051	−0.523 ***
	(0.54)	(−3.25)
1._cat#c.h	−0.366 ***	−0.389 ***
	(−4.92)	(−3.90)
Control variables	Y	Y
F	11.51	7.281
R-squared	0.284	0.201
Observations	270	270

Note: *** refers to significance at the level of 1%, respectively.

## Data Availability

The data presented in this study are openly available in provincial statistical yearbooks of China, the China Statistical Yearbook, China Health Statistics Yearbook, China Environmental Statistics Yearbook, available at website: https://data.cnki.net/Yearbook/Navi?type=type&code=A, (accessed on 10 November 2021) or http://olap.epsnet.com.cn (accessed on 10 November 2021); China Stock Market and Accounting Research Database, available at website: http://www.gtarsc.com.bbb.hejiantv.cn/#/index (accessed on 10 November 2021); China Economic Network Statistical database, available at website: https://db--cei--cn--dwjj.h.gou5juan.com/ (accessed on 10 November 2021); meteorological monitoring data of Columbia University (Socioeconomic Data and Applications Center (sedac)), available at website: https://sedac.ciesin.columbia.edu/data/set/sdei-global-annual-gwr-pm2-5-modis-misr-seawifs-aod/data-download (accessed on 10 November 2021).
